# Supplementary material for: Single-site decorated copper enables energy- and carbon-efficient CO2 methanation in acidic conditions
Source: Nat Commun. 2023 Jun 7;14:3314. doi: 10.1038/s41467-023-38935-2 (PMC10247791; doi:10.1038/s41467-023-38935-2)
Supplement: Supplementary file 1 — Supplementary Information [file 41467_2023_38935_MOESM1_ESM.pdf]

Supplementary information

Single-site Decorated Copper Enables Energy- and Carbon-efficient CO_2 Methanation in Acidic Conditions

Mengyang Fan[1†], Rui Kai Miao[1†], Pengfei Ou[2†], Yi Xu[1†], Zih-Yi Lin[3], Tsung-Ju Lee[3], Sung-Fu Hung[3], Ke Xie[2], Jianan Erick Huang[2], Weiyan Ni[2], Jun Li[1], Yong Zhao[1], Adnan Ozden[1], Colin P O'Brien[1], Yuanjun Chen[2], Yurou Celine Xiao[1], Shijie Liu[1], Joshua Wicks[2], Xue Wang[2], Jehad Abed[2], Erfan Shirzadi[2], Edward H. Sargent[2]* & David Sinton[1]*

[1]*Department of Mechanical and Industrial Engineering, University of Toronto, 5 King's College Road, Toronto, Ontario, M5S 3G8, Canada.*

[2]*Department of Electrical and Computer Engineering, University of Toronto, 10 King's College Road, Toronto, Ontario, M5S 3G4, Canada.*

[3]*Department of Applied Chemistry, National Yang Ming Chiao Tung University, Hsinchu, Taiwan*

[†]*These authors contributed equally.*

**Corresponding email:* ted.sargent@utoronto.ca *and* sinton@mie.utoronto.ca

Supplementary Note 1 | Energy assessment of the CO_2R system. Energy assessment was performed using an energy assessment model adopted from that reported in ref[1,2]. This section provides a brief description of the model along with the assumptions. The model uses the performance metrics of the CO_2R system to estimate the energy intensity of producing CH_4 from CO_2. These metrics include full-cell potential, single pass CO_2 conversion efficiency, Faradaic efficiency and current density – all towards CH_4. The energy assessment was performed at various flow rates of feedstock CO_2 by using the associated performance metrics. H_2 produced from competing hydrogen evolution reaction (HER) was considered the only by-product at the cathodic stream, besides the unreacted CO_2 and CH_4. Meanwhile, O_2 produced from oxygen evolution reaction (OER) was considered only product at the anodic stream, as the system enables local regeneration of CO_2, and thereby blocking the CO_2 crossover to the anodic stream. To recover CH_4 from the unreacted CO_2 and H_2, a pressure swing adsorption (PSA) module is modelled to be at the cathodic downstream. The CO_2 recovered from the cathodic stream is modelled to be recirculated to the cathode inlet for utilization in CO_2R. Further details of the model and assumptions can be found in ref.[1,2]. An example energy cost calculation for the electrolyzer electricity, cathode separation, and anode separation is provided in Supplementary Note 2.

Supplementary Note 2 | Example energy cost calculation for CO_2-to-CH_4 conversion. This section describes the energy cost associated with electrolyzer electricity, cathode separation, and anode separation in the acidic BP-MEA system. The calculation details are based on the performance metrics that enable the lowest energy intensity of producing CH_4 in the acidic BP-MEA system. These metrics include a full-cell voltage of 3.56 V, a CH_4 Faradaic efficiency of 71%, a CO_2-to-CH_4 SPCE of 14%, and a current density of 100 mA cm^{-2} (**Table 1**).

Electrolyzer electricity. We first calculate the production capacity of CH_4 on a molar basis for a constant production capacity of 100 tonne CH_4 per day.

$$Production\ rate\ \left[\frac{mol}{s}\right] = \frac{Production\ \left[\frac{g}{day}\right]}{molecular\ weight_{CH4}\ \left[\frac{g}{mol}\right] \times 86400\ \left[\frac{s}{day}\right]} \quad (1a)$$

$$Production\ rate\ \left[\frac{mol}{s}\right] = \frac{100 \times \frac{10^6 g}{day}}{\frac{16\ g}{mol} \times \frac{86400 s}{day}} = 72.3 \frac{mol}{s} \quad (1b)$$

Then, we calculate the current needed to electroproduce CH_4 at this productivity by using a CH_4 FE of 71%:

$$Total\ current\ required\ [A] = \frac{production\ rate\ \left[\frac{mol}{s}\right] \times No.e^{-}\ transferred \times Faraday's\ constant}{FE_{CH_4}} \quad (2a)$$

$$Total\ current\ needed\ [A] = \frac{72.3 \frac{mol}{s} \times 8 \times 96485 \frac{sA}{mol}}{71\%} = 78\ 642\ 573\ A \quad (2b)$$

Then, we calculate the power consumption by multiplying the current with the cell potential of 3.56 V:

$$\begin{gathered}\textit{Power Consumed}\ [W] = \\ \textit{Total current needed}\ [A] \times \textit{Cell voltage}\ [V] = 78\,641\,526\ A \times 3.56\ V = 279\,968\ kW\end{gathered} \tag{3}$$

Then, we find the energy required to meet the production capacity of 100 tonne per day:

$$\begin{gathered}\textit{Electricity Energy Requirement}\ \left[\frac{GJ}{tonne_{CH_4}}\right] = \\ \frac{\textit{Power Consumed}\ [W] \times 24[h]}{\textit{daily production}\ [tonne]} = \frac{279\,968\ kW \times 24\ h \times 0.0036\ GJ/kWh}{100\ tonne} = \mathbf{241.9}\ \frac{\mathbf{GJ}}{\mathbf{tonne_{CH_4}}}\end{gathered} \tag{4}$$

Cathode separation. To estimate the energy cost associated with the recovery of CH_4 from the cathodic downstream, we assumed a pressure swing adsorption (PSA) separation module. The capital and operational energy cost of the PSA unit is hinged on a model built for biogas separation. The cathodic stream considers the presence of CH_4, unreacted CO_2 and H_2 at the cathodic downstream. The model takes a cost of $1 989 043 into account for a flow rate of 1000 $m^3\ h^{-1}$ and uses a scaling factor of 0.7 and energy demand of 0.25 kWh m^{-3}. According to this, we estimate the energy requirement:

$$\textit{PSA operating energy}\ \left[\frac{\text{kWh}}{tonne_{CH_4}}\right] = 0.25\ \frac{kWh}{m^3} \times \textit{flow rate}\left[\frac{m^3}{h}\right] \times 24\frac{h}{day} \tag{5a}$$

Prior to using this correlation, we estimate the flow rate at the cathodic stream. Assuming operation under the ideal conditions, we estimated the flow rate of CH_4:

$$\textit{Output}\ CH_4\ \textit{flow rate}\ \left[\frac{m^3}{h}\right] = \frac{100 \times 10^6\ \frac{g}{day} \times 8.314\ Jmol^{-1}K^{-1} \times 298K}{16\frac{g}{mol} \times 101\,300\ Pa \times 24\frac{h}{day}} = 6369\ \frac{m^3}{h} \tag{6}$$

We then estimate the flow rates of CO_2, CH_4, and H_2 at the cathodic stream. The flow rate of CO_2 at the cathodic stream is calculated by using the experimentally achieved SPCE at a constant pressure. We note that this SPCE relates to the amount of CO_2 converted to that of unreacted CO_2. We then calculate the output CO_2 flow rate considering a SPCE of 14%:

$$\textit{Output}\ CO_2\ \textit{flow rate}\ \left[\frac{m^3}{h}\right] =$$

$$CH_4\ \textit{flow rate}\ \left[\frac{m^3}{h}\right] \times \textit{molar ratio}\ \left[\frac{CO_2}{CH_4}\right] \times \left(\frac{1 - SPCE}{SPCE}\right) \tag{7a}$$

$$\textit{Output}\ CO_2\ \textit{flow rate}\ \left[\frac{m^3}{h}\right] = 6369\frac{m^3}{h} \times 1 \times \frac{1 - 0.14}{0.14} = 39124\ \frac{m^3}{h} \tag{7b}$$

We then calculate the current toward H_2:

$$Current\ toward\ H_2[A] = Total\ current\ needed[A] \times \frac{1 - FE_{methane}}{1} \quad (8a)$$

$$Current\ toward\ H_2[A] = 78\ 642\ 573\ A \times \frac{1 - 0.71}{1} = 22\ 806\ 346\ A \quad (8b)$$

The production rate of H_2 can also be calculated as follows.

$$H_2\ production\ \left[\frac{mol}{h}\right] = \frac{Current\ toward\ H_2[A] \times 3600\frac{s}{h}}{2\frac{electrons}{H_2\ product} \times Faraday's\ constant} \quad (9a)$$

$$H_2\ production\ \left[\frac{mol}{h}\right] = \frac{22\ 806\ 346\ A \times 3600\frac{s}{hour}}{2\frac{electrons}{H_2\ product} \times 96485\frac{sA}{mol}} = 425\ 463\ \frac{mol}{h} \quad (9b)$$

Assuming an ideal gas under standard conditions, the flow rate of H_2 can be calculated:

$$flow\ rate_{H2}\left[\frac{m^3}{h}\right] = \frac{H_2\ production\left[\frac{mol}{h}\right] \times 8.314\frac{J}{mol \times K} \times 298K}{101.3 \times 10^3 Pa} \quad (10a)$$

$$flow\ rate_{H2}\left[\frac{m^3}{h}\right] = \frac{425\ 463\frac{mol}{h} \times 8.314\frac{J}{mol \times K} \times 298K}{101.3 \times 10^3 Pa} = 10\ 405\ \frac{m^3}{h} \quad (10b)$$

The total flow rate of the cathodic stream can then be calculated by using the flow rates of CH_4, unreacted CO_2, and H_2 using:

$$flow\ rate\ \left[\frac{m^3}{h}\right] = (6369 + 39124 + 10405)\frac{m^3}{h} = 55\ 898\frac{m^3}{h} \quad (11)$$

Next, we can calculate the energy required per tonne of CH_4 produced by using:

$$PSA\ Energy\ \left[\frac{GJ}{tonne_{CH_4}}\right] = 0.25\ \frac{kWh}{m^3} \times 55\ 898\frac{m^3}{h} \times 24\frac{h}{day} \times \frac{0.0036\ GJ\ kWh^{-1}}{100\ tonne_{CH_4}}$$
$$= \mathbf{12.1}\ \frac{\mathbf{GJ}}{\mathbf{tonne_{CH_4}}} \quad (12)$$

In alkaline flow cell and neutral MEA, CO_2 loss to crossover and/or carbonates reduces cathodic CO_2 flow rate, and therefore, must be considered to correctly estimate CO_2 flowrate at cathodic outlet. A crossover factor (CO_2 crossover/CO_2 reduced) of 4 and a carbonate loss factor (CO_2 loss to carbonate/CO_2 reduced) of 20 are considered for MEA and alkaline flow cell, respectively. The cathodic CO_2 flow rate can be found using the equation below[3,4]:

$$Output\ CO_2\ flow\ rate\ \left[\frac{m^3}{h}\right] =$$

$$\frac{CH_4\ flow\ rate\ \left[\frac{m^3}{h}\right] \times molar\ ratio\ \left[\frac{CO_2}{CH_4}\right]}{\left(\frac{SPCE}{\frac{1}{loss\ \ factor + 1}}\right)} \times \left(1 - \frac{SPCE}{\frac{1}{loss\ \ factor + 1}}\right) \quad (13)$$

Supplementary Note 3 | CO_2 loss to carbonate and energy cost calculation for CO_2 regeneration in alkaline flow cells. In alkaline flow cell, input CO_2 is rapidly consumed by the excess of hydroxides in the catholytes according to Eq. 1.

$$CO_2 + 2OH^- \rightarrow CO_3^{2-} + H_2O \quad \text{Eq. 1}$$

Prior reports had found that more than 20 CO_2 is lost to carbonates for every CO_2 reduced to product[3,4]. Current regeneration of CO_2 from carbonate solutions relies on calcium caustic loop, which requires a thermal energy input of 5.25 GJ/tonne CO_2[5]. CO_2 loss to carbonates in alkaline electrolytes per tonne of CH_4 can be calculated as:

$$CO_2\ loss \left[\frac{tonne\ CO_2}{tonne\ CH_4}\right] =$$

$$1\ tonne_{CH_4} \times \frac{molecular\ weight_{CO_2}\left[\frac{g}{mol}\right]}{molecular\ weight_{CH_4}\left[\frac{g}{mol}\right]} \times molar\ ratio\ \left[\frac{CO_2}{CH_4}\right] \times carbonate\ loss\ factor =$$

$$1\ tonne_{CH_4} \times \frac{44\frac{g}{mol}}{16\frac{g}{mol}} \times 1 \times 20 = \mathbf{55}\ \frac{\boldsymbol{tonne\ CO_2}}{\boldsymbol{tonne\ CH_4}} \quad (14)$$

The thermal energy required to regenerate CO_2 from carbonate solutions can be found:

$$Carbonate\ regeneration\ \left[\frac{GJ}{tonne_{CH_4}}\right] = 5.25\ \left[\frac{GJ}{tonne\ CO_2}\right] \times CO_2\ loss \left[\frac{tonne\ CO_2}{tonne\ CH_4}\right]$$

$$= 5.25\ \frac{GJ}{tonne\ CO_2} \times 55\ \frac{tonne\ CO_2}{tonne\ CH_4} = \mathbf{288.8}\ \frac{\boldsymbol{GJ}}{\boldsymbol{tonne_{CH_4}}} \quad (15)$$

Recovering CO_2 from carbonate solutions costs 288.8 GJ/tonne CH_4 – more than five times of CH_4 heating value. Considering calcium caustic loop relies on high grade (>900°C) thermal input, this implies that regenerating loss CO_2 from alkaline flow cell requires consuming five times more fuels (typically CH_4) than it is produced through electrolysis, making these systems unviable going forward.

Supplementary Note 4 | CO_2 loss to crossover and energy cost calculation for CO_2/O_2 separation in neutral MEAs. CO_2 loss to carbonate is less severe in neutral MEAs compared to alkaline flow cell. In neutral MEAs, both CO_2R and the competing HER generates hydroxides, and these hydroxides consumes CO_2 to form carbonates. Negatively charged carbonates migrate through the anion-selective membrane and revert back to CO_2 at the anode where oxygen evolution takes place, resulting a mixture of CO_2/O_2. This CO_2/O_2 mixture could not be directly recirculated back to the cathode; rather, further separation is necessary. For CO_2-to-CH_4 conversion, producing each CH_4 (or consuming each CO_2) generates eight hydroxides (Eq. 2). These eight hydroxides consume at least four CO_2 (Eq. 3 or Eq. 4), limiting the maximum single-pass CO_2 conversion efficiency to 20% in neutral MEAs.

$$CO_2 + 6H_2O + 8e^- \rightarrow CH_4 + 8OH^- \quad \text{Eq. 2}$$

$$8CO_2 + 8OH^- \rightarrow 8HCO_3^{2-} \quad \text{Eq. 3}$$

$$4CO_2 + 8OH^- \rightarrow 4CO_3^{2-} + 4H_2O \quad \text{Eq. 4}$$

Separating this CO_2/O_2 mixture currently require amine-based capture estimated at 4 GJ/tonne CO_2[6]. CO_2 crossover per tonne of CH_4 (ideal case: 100% CH_4 FE) can be calculated as:

$$CO_2\ loss\left[\frac{tonne\ CO_2}{tonne\ CH_4}\right] =$$

$$\frac{1\ tonne_{CH_4} \times \dfrac{molecular\ weight_{CO_2}\left[\frac{g}{mol}\right]}{molecular\ weight_{CH_4}\left[\frac{g}{mol}\right]} \times molar\ ratio\ \left[\frac{CO_2}{CH_4}\right] \times crossover\ factor}{FE_{methane}} =$$

$$\frac{1\ tonne_{CH_4} \times \dfrac{44\frac{g}{mol}}{16\frac{g}{mol}} \times 1 \times 4}{1} = \mathbf{11}\ \frac{\mathbf{tonne\ CO_2}}{\mathbf{tonne\ CH_4}} \quad (16)$$

The energy input to separate CO_2 from anodic mixture can be found:

$$Anode\ separation\left[\frac{GJ}{tonne_{CH_4}}\right] = 4\ \left[\frac{GJ}{tonne\ CO_2}\right] \times CO_2\ loss\left[\frac{tonne\ CO_2}{tonne\ CH_4}\right]$$

$$= 4\ \frac{GJ}{tonne\ CO_2} \times 11\ \frac{tonne\ CO_2}{tonne\ CH_4} = \mathbf{44}\ \frac{\mathbf{GJ}}{\mathbf{tonne_{CH_4}}} \quad (17)$$

Therefore, at 100% CH_4 FE, separating CO_2 from anodic CO_2/O_2 mixture requires 44 GJ/tonne CH_4. State-of-the-art CH_4 producing CO_2 electrolyzers have selectivity ranged from 60-80%, corresponding to an anode separation cost of 55-73 GJ/tonne CH_4. This energy exceeds CH_4 heating value, implying that more thermal energy is required to recover CO_2 in neutral MEAs than that can be acquired from the produced CH_4.

Table S1 Electrolyzer energy distribution comparison of different systems

Parameters	CO_2-to-CH_4 (Scenario 1)[7]	CO_2-to-CH_4 (Scenario 2)[8]	CO_2-to-CH_4 (This work)	
Systems	Neutral MEA	Alkaline flow cell	Acidic microchannel-MEA	
Cell voltage (V)	4.0	4.0	3.56	4.12
Faradaic efficiency (%)	62	82	71	64
Current density (mA cm^{-2})	220	480	100	200
Single pass conversion CH_4	1.6	3.7	14	14
Electrolyzer electricity	311.2	235.3	241.9	310.5
Cathode separation (GJ/tonne)	20.6	3.0	12.1	12.9
Anode separation (GJ/tonne)	71.0	0.0	0.0	0
Carbonate regeneration	0.0	288.8	0.0	0
**Overall energy (GJ/tonne)**	**402.8**	**527.1**	**254.0**	**323.4**

High energy efficiency scenario (Flow rate of CO_2 in Scenario 1. is 80 sccm, flow rate of CO_2 in Scenario 2 is 20 sccm, and flow rate of CO_2 in this work is 1.4 sccm) and performance metrics used as the input corresponding to those achieved with the flow rates listed above.

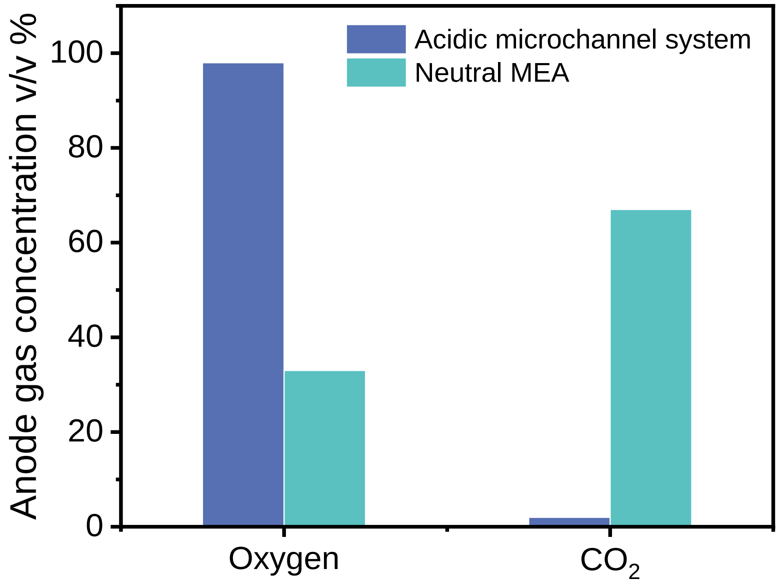

Figure S1 Comparison of anodic CO_2 loss. The neutral MEA used an AEM as membrane, and circulated 10 mM $KHCO_3$ as anolyte. The acidic MEA used the AEM/channeled CEM combination and circulated 5 mM H_2SO_4 as anolyte. The applied current density was 100 mA cm^{-2}.

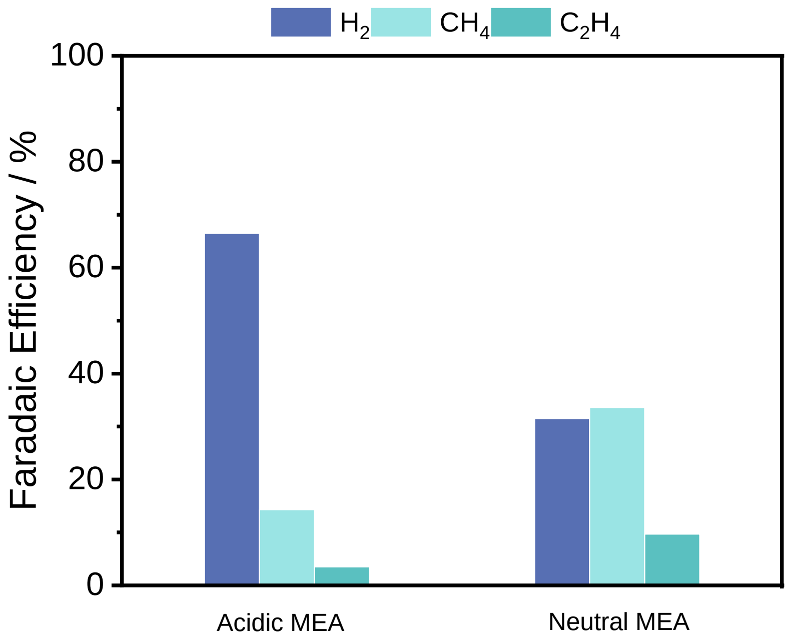

Figure S2 CO_2R performance comparison in different system. The catalysts used here were all CuPc with a loading of 0.1 mg cm^{-2}. The CO_2R performances were tested under the same current density of 100 mA cm^{-2}. In the acidic MEA, 5 mM H_2SO_4 was used as the anolyte. In the neutral MEA, 10 mM $KHCO_3$ was used as anolyte.

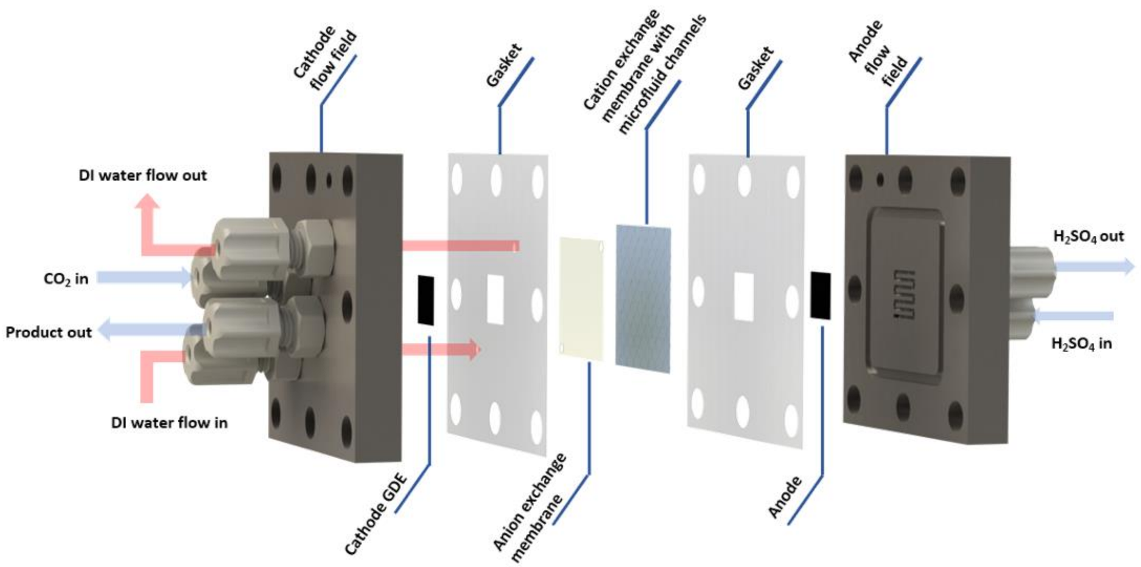

Figure S3 Schematic illustration of the acidic channeled-MEA system. The channeled CEM has a pore path of 75µm. The anolyte was circulated with 5 mM H_2SO_4.

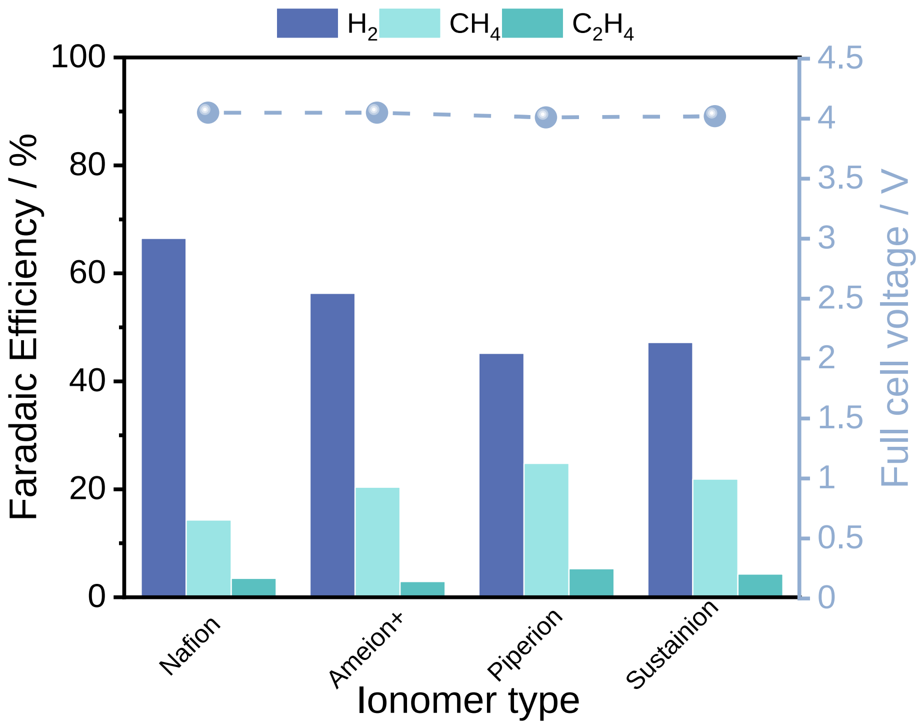

Figure S4 CO_2R performance comparison of different type of ionomer binders. The catalysts used here were all CuPc with a loading of 0.1 mg cm^{-2}. The CO_2R performances were tested under the same current density of 100 mA cm^{-2}. The cation exchange Nafion binder shows slightly higher H_2 FE compared to the other anion exchange binders.

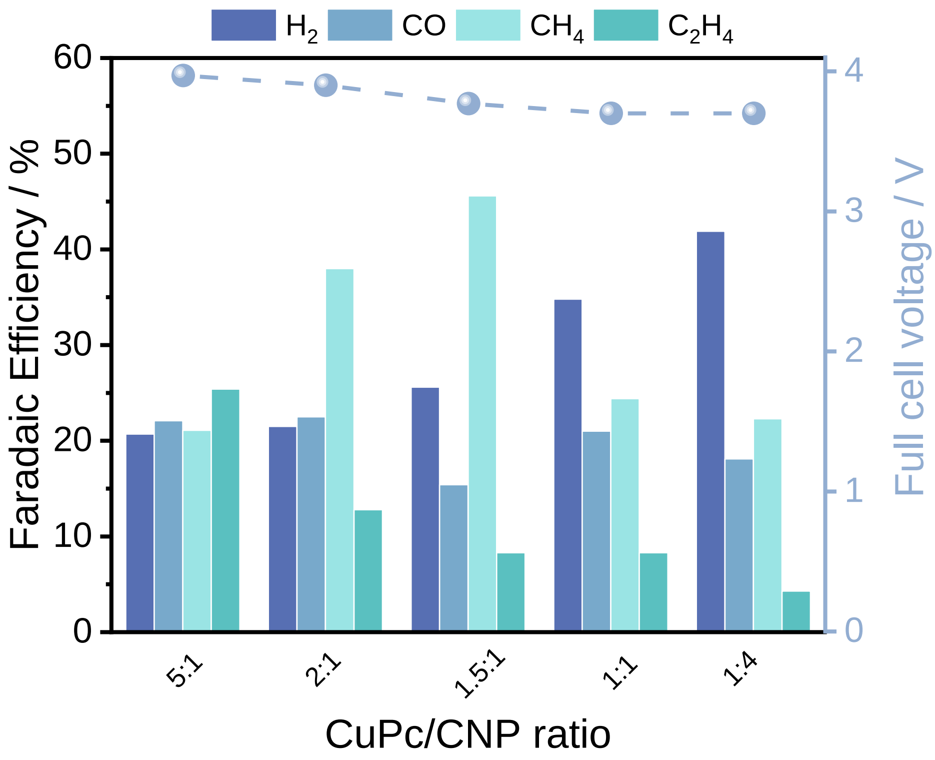

Figure S5 Product distribution at different CuPc/CNP ratios. Ionomer binder used in all cases was PiperIon. The tests were performed at a constant current of 100 mA cm^{-2}. 5 mM H_2SO_4 was circulated as analyte.

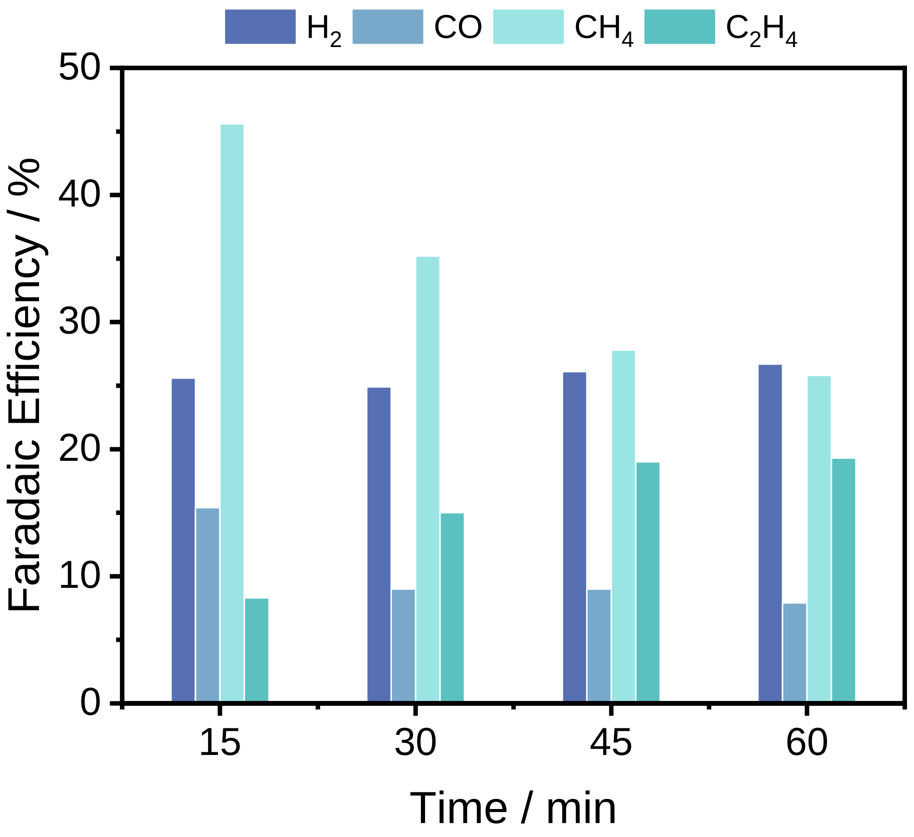

Figure S6 FE performance of CuPc/CNP. The optimal ratio of 1.5:1 was used for CuPc/CNP catalyst. The tests were performed at a constant current of 100 mA cm^{-2}. 5 mM H_2SO_4 was circulated as anolyte.

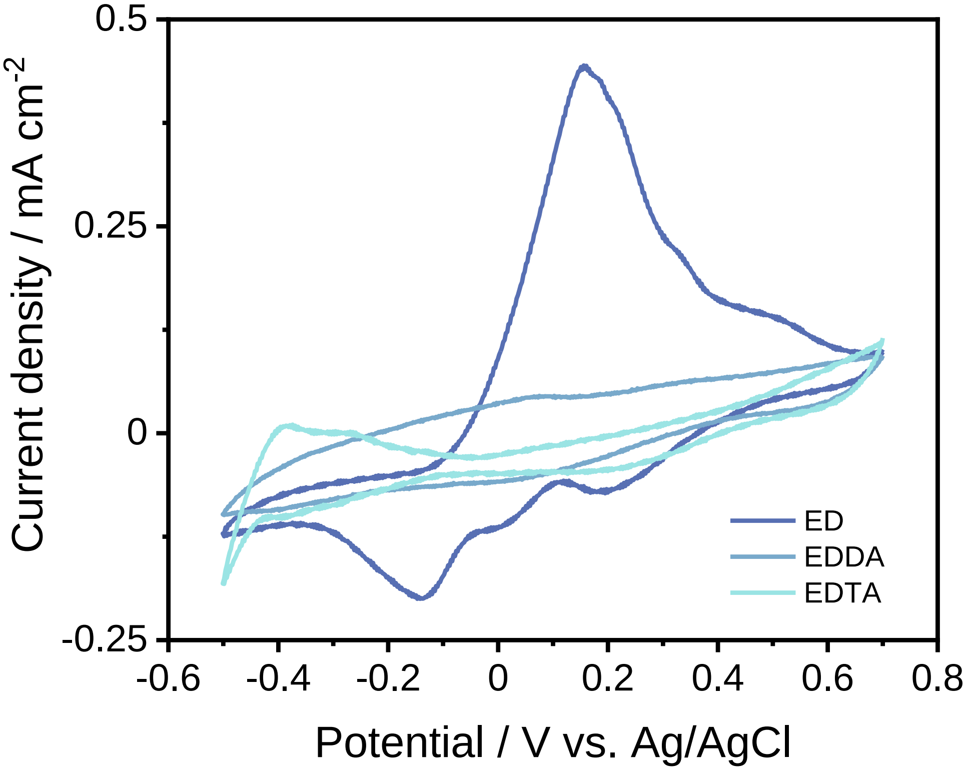

Figure S7 CV characterization of Cu redox peaks. Ethylenediamine (ED), ethylenediamine-N,N′-diacetic acid (EDDA), and ethylenediaminetetraacetic acid (EDTA) provide bidentate, tetradentate and hexadentate coordinated sites, respectively. 1 mM $CuSO_4$ was added to 10 mM ED, EDDA and EDTA, respectively, to form the Cu complex by bonding Cu ion with multidentate coordination sites. The EDDA presented a much lower Cu redox peak compared with the ED, indicating more intensive bonds between Cu and EDDA. For EDTA, barely Cu redox peaks were observed, demonstrating a stronger interaction between Cu and EDTA. Pt foil and Pt gauze were used as working and counter electrodes. The tests were performed at a scan rate of 100 mV s^{-1}.

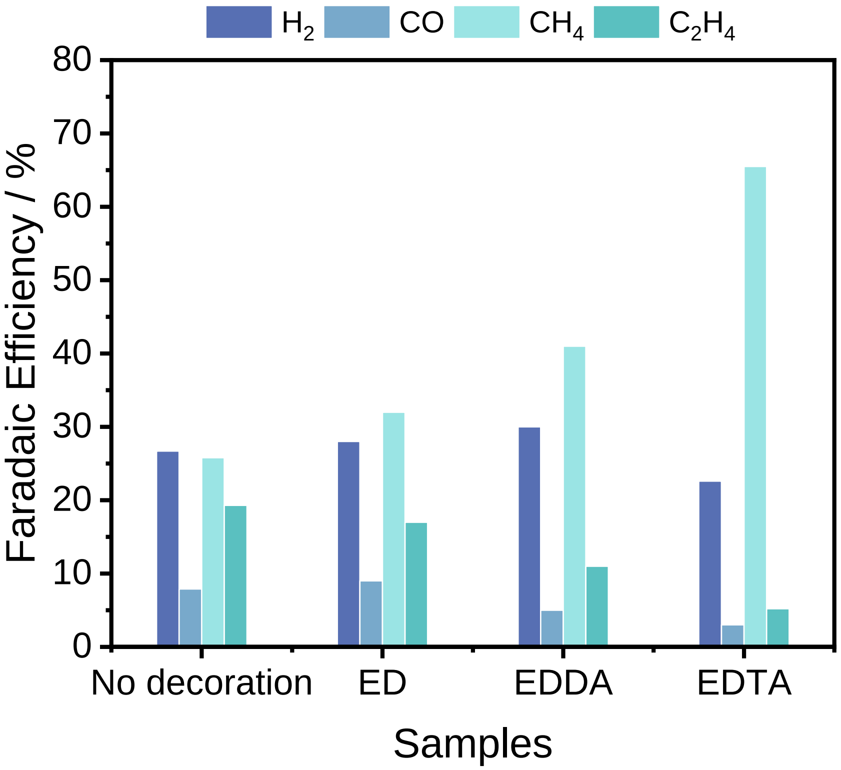

Figure S8 FE performance of CuPc/CNP samples with and without decoration at the initial 1 hour of electrolysis. The optimal ratio of 1.5:1 was used for CuPc/CNP catalyst. The ratio between CuPc/CNP and each molecule keep constant at 1:4. The tests were performed at a constant current of 100 mA cm^{-2}. 5 mM H_2SO_4 was circulated as anolyte.

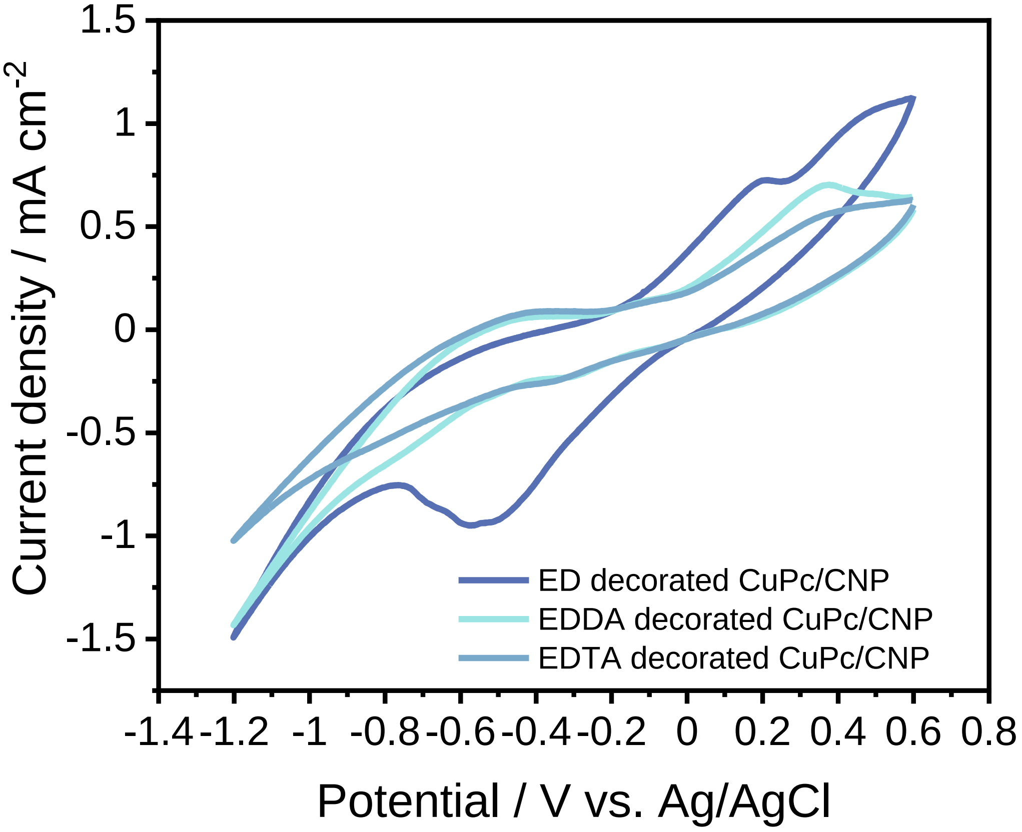

Figure S9 CV characterization of Cu redox peaks. The ratio between CuPc/CNP and each molecule keep constant at 1:4. We fabricated the ED, EDDA and EDTA decorated CuPc/CNP composite catalysts by spray-coating the mixture onto the gas diffusion layers (GDLs). The tests were performed at a scan rate of 100 mV s^{-1} in a 5 mM H_2SO_4 electrolyte. The molecules decorated CuPc/CNP GDLs were used as the working electrode. ED decorated sample showed an obvious Cu redox peak, indicating a weak interaction between Cu(II) and ED. EDTA decorated samples presented minimal Cu redox peaks, indicating a strong interaction between Cu and hexadentate coordination sites that constrain Cu(II) from the CuPc/CNP precursor.

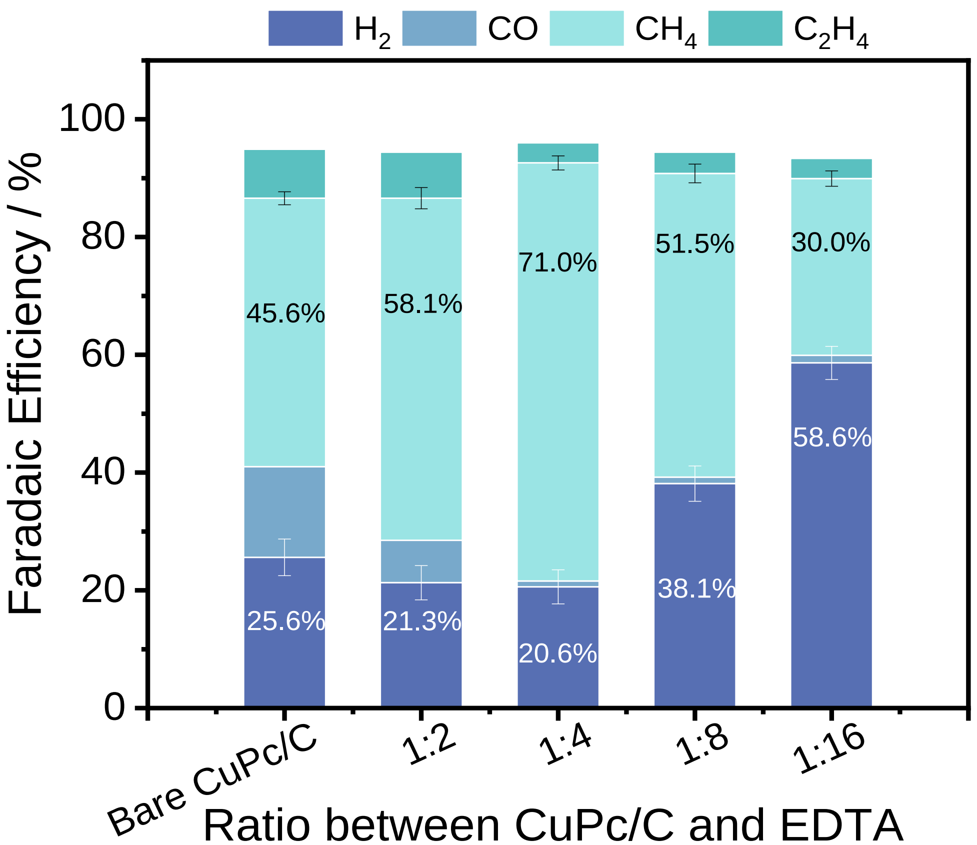

Figure S10 Product distribution of the CuPc/CNP/EDTA samples with various EDTA mass loadings. The CuPc/CNP ratio was constant at 1.5:1. The applied current density was at a constant value of 100 mA cm^{-2}. Values are means, and error bars indicate SD (n = 3 replicates)

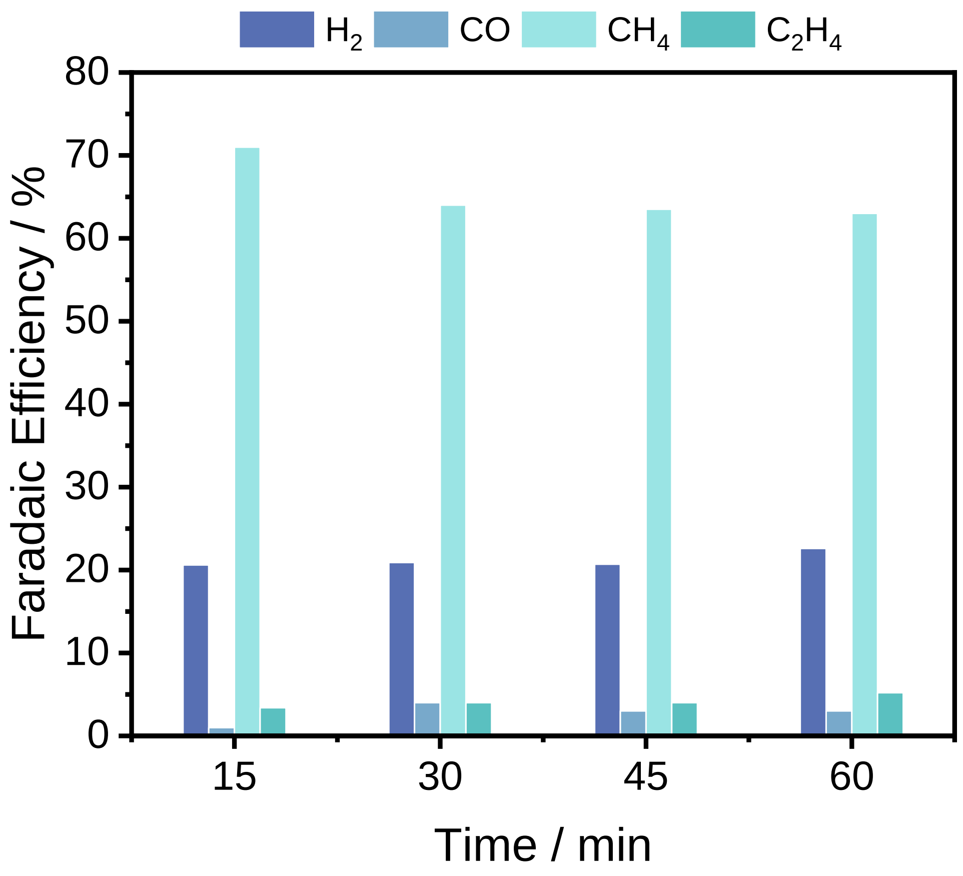

Figure S11 FE performance of EDTA decorated CuPc/CNP. The ratio between EDTA and CuPc/CNP was 1:4. The tests were performed at a constant current of 100 mA cm^{-2}. 5 mM H_2SO_4 was circulated as anolyte.

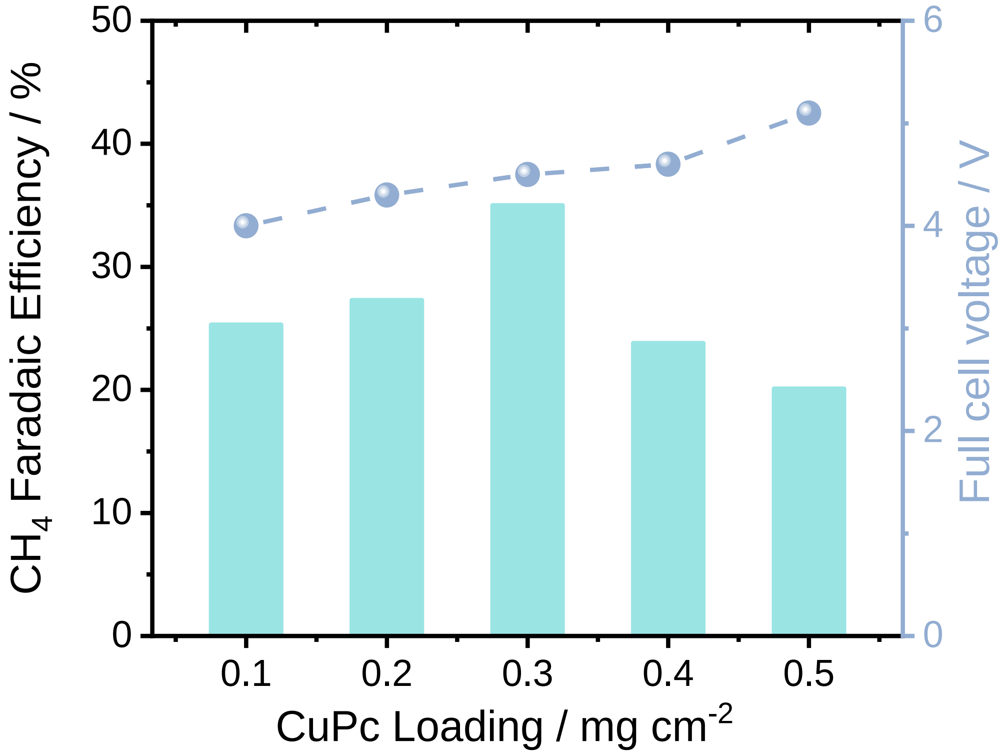

Figure S12 CH_4 selectivity of the pristine CuPc. The CuPc loadings ranged from 0.1 to 0.5 mg cm^{-2}. There is no CNP and EDTA addition in this pristine CuPc. Ionomer binder was PiperIon. The tests were performed at a constant current of 100 mA cm^{-2}. 5 mM H_2SO_4 was circulated as anolyte.

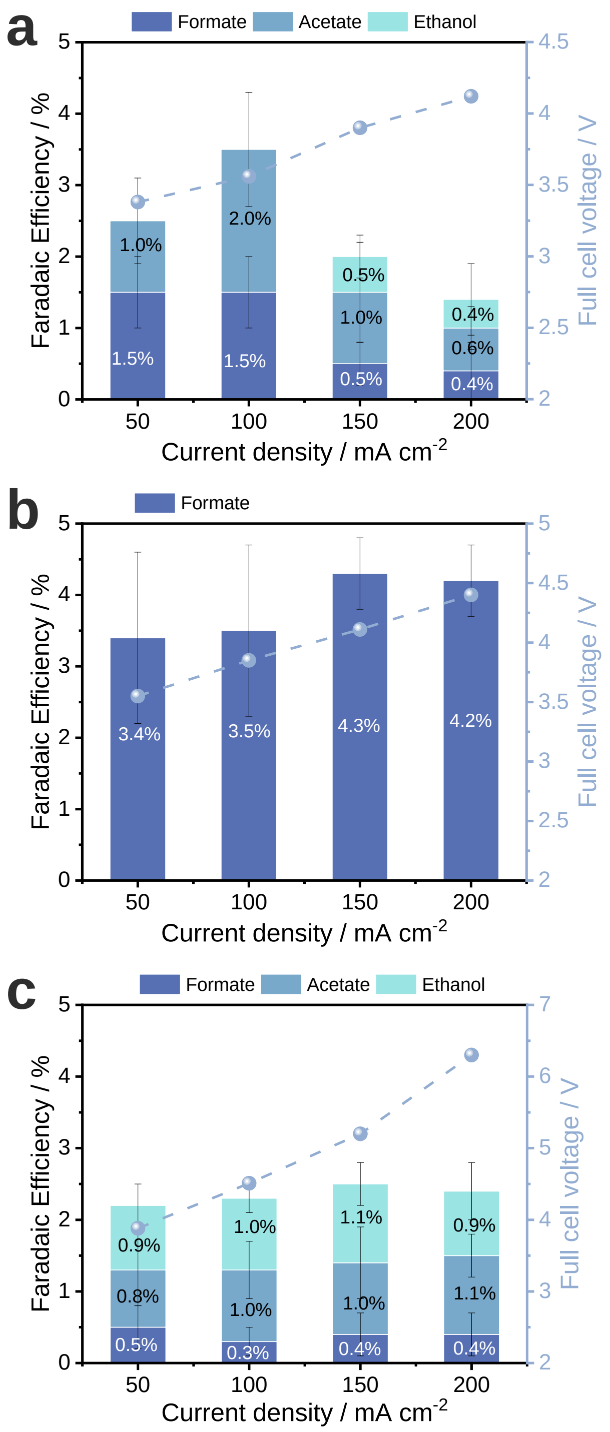

Figure S13 Liquid products distribution of different samples. (a) EDTA/CuPc/CNP, (b) EDTA/CNP and (c) EDTA/CuPc at current range from 50 to 200 mA cm^{-2}. Values are means, and error bars indicate SD (n = 3 replicates)

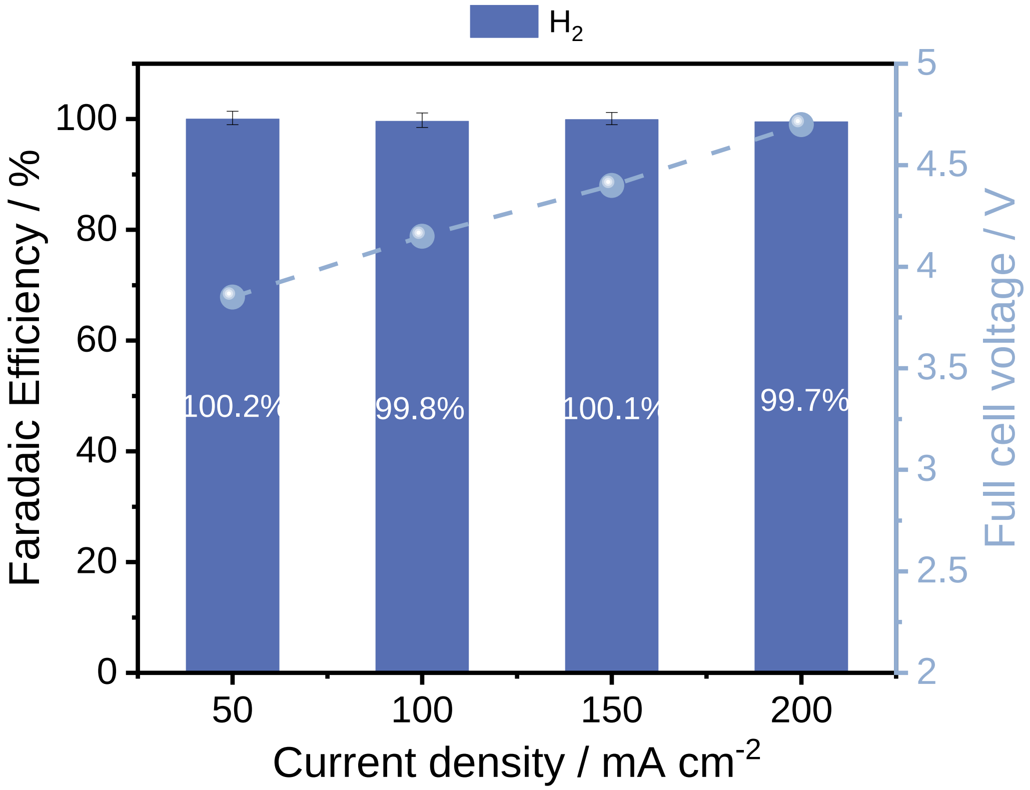

Figure S14 FE performance under Ar conditions. The samples EDTA/CuPc/CNP was used as cathode and reduction reaction was performed at different current densities ranging from 50 to 200 mA cm^{-2}. The Ar flow rate was 20 sccm cm^{-2}. Values are means, and error bars indicate SD (n = 3 replicates)

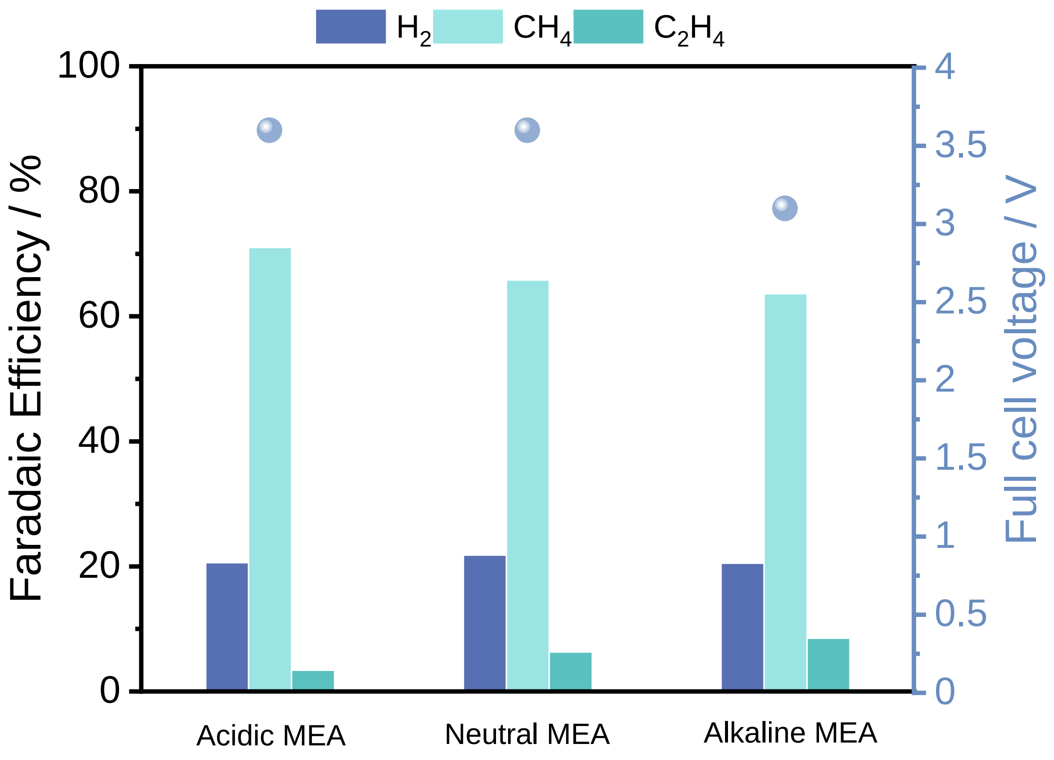

Figure S15 CO_2R performance comparison of acidic, neutral and alkaline MEA systems. The tests were performed at a constant current of 100 mA cm^{-2}. The cathode used was the optimized EDTA/CuPc/CNP sample.

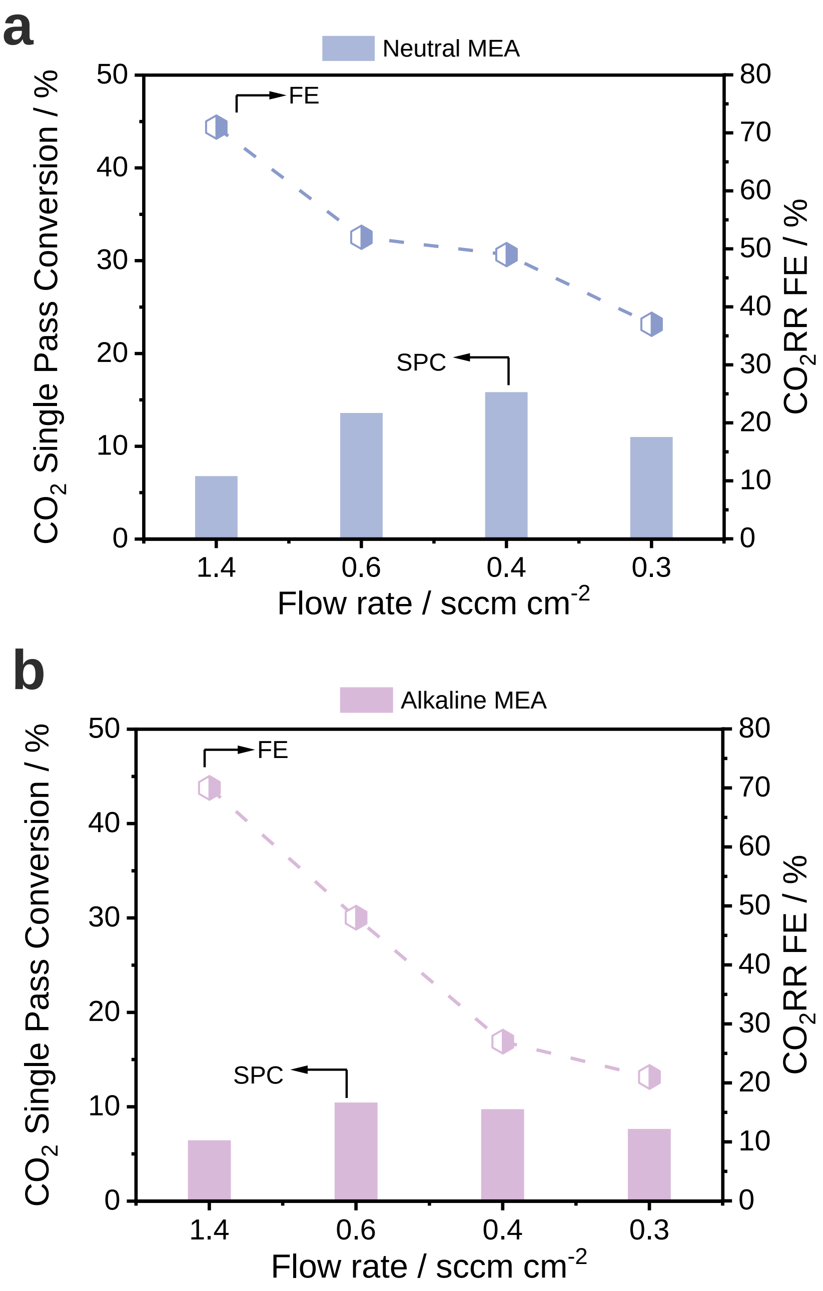

Figure S16 Single pass conversion of CO_2 at different flow rates. (a) In the neutral MEA system, a neutral 0.5 M $KHCO_3$ was used as the anolyte and (b) In the alkaline MEA system, 0.5M KOH was used as the anolyte. The anolyte flow rate was 5mL min^{-1}. The cathode and anode were separated by an anion exchange membrane. The SPC results were obtained at a constant current density of 100 mA cm^{-2}.

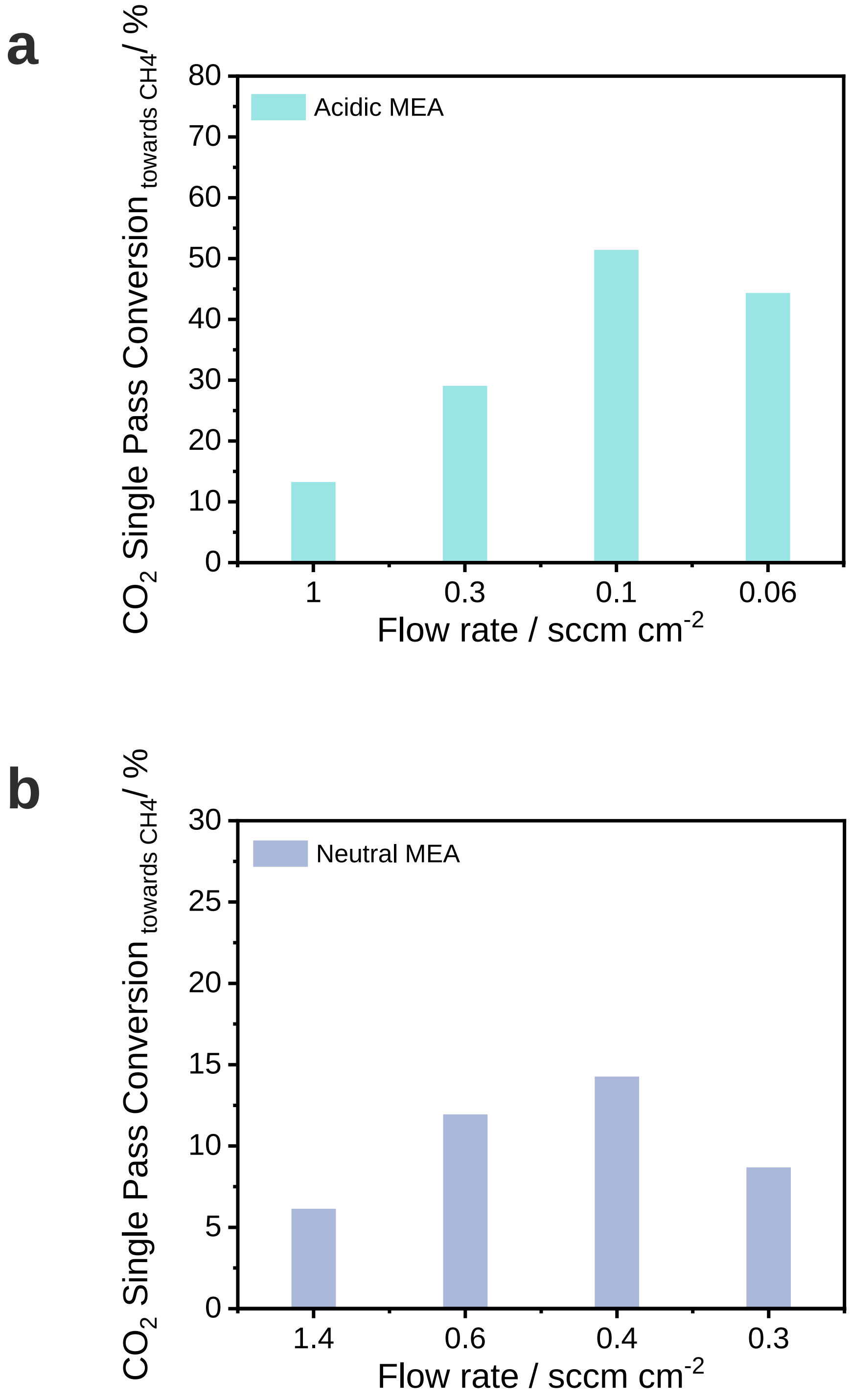

Figure S17 CO_2 single pass conversion towards CH_4. (a) Acidic MEA system, (b) neutral MEA system. In the neutral system, 0.5 M $KHCO_3$ was used as anolyte with a constant flow rate of 5 mL min^{-1}. An anion exchange membrane was used in the neutral MEA to separate the cathode and anode. The SPC results were obtained at a constant current density of 100 mA cm^{-2}.

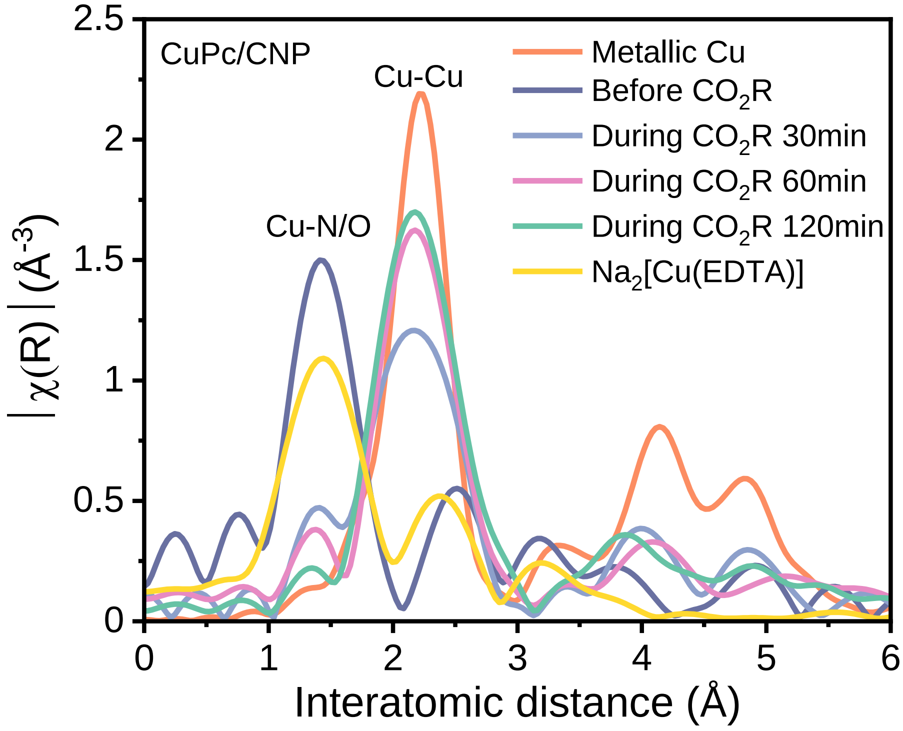

Figure S18 *In-situ* Fourier-transformed Cu EXAFS spectra of CuPc/CNP. Spectra collected at 100 mA cm^{-2} under CO_2R conditions. Metallic Cu, and Na_2[Cu(EDTA)] samples are shown as references.

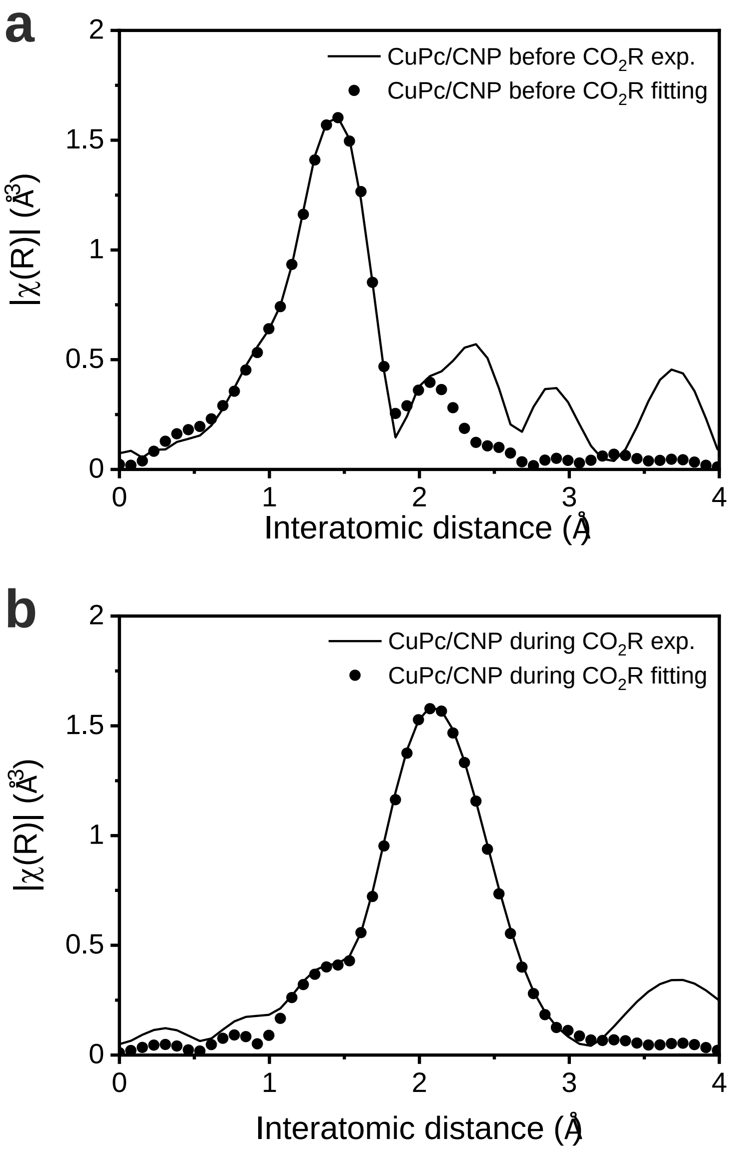

Figure S19 Fourier-transformed Cu K-edge EXAFS spectra fitting lines for the CuPc/CNP sample. Fitting lines for samples (a) before and (b) during CO_2R. The spectrum was taken under 100 mA cm^{-2} during 120 min of CO_2R.

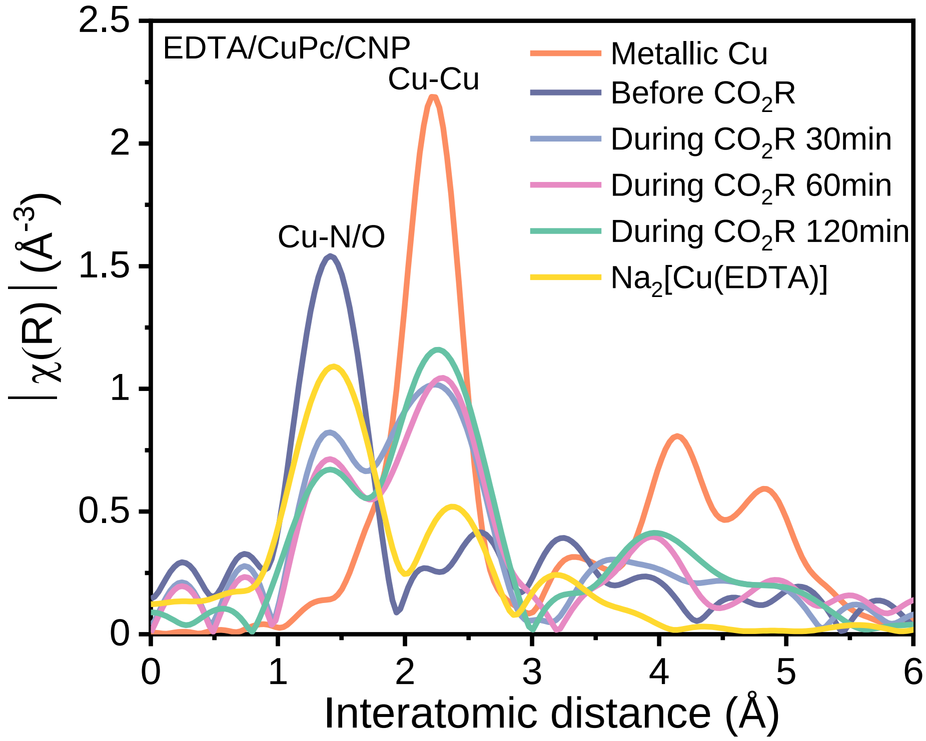

Figure S20 *In-situ* Fourier-transformed Cu EXAFS spectra of EDTA decorated CuPc/CNP. Spectra collected at 100 mA cm^{-2} under CO_2R conditions. Metallic Cu, and $Na_2[Cu(EDTA)]$ samples are shown as references.

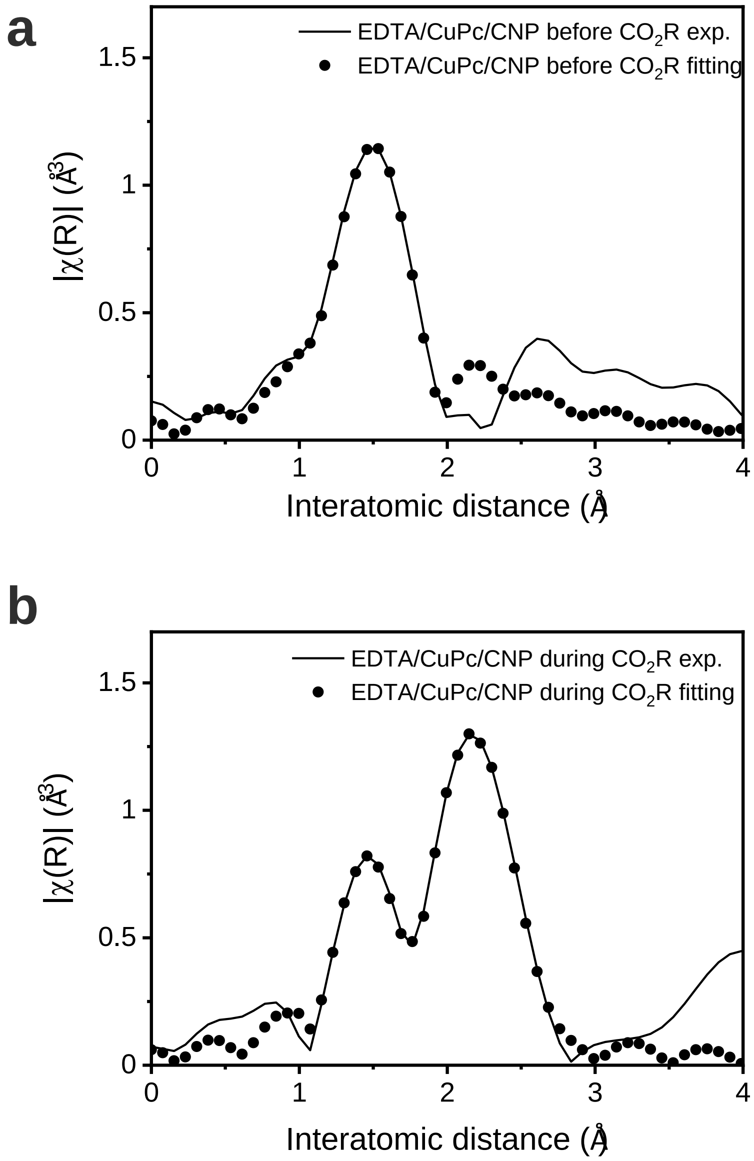

Figure S21 Fourier-transformed Cu K-edge EXAFS spectra fitting lines for the EDTA decorated CuPc/CNP sample. Fitting lines for samples (a) before and (b) during CO_2R. The spectrum was taken under 100 mA cm^{-2} during 120 min of CO_2R.

Table S2 Fitting parameters for the samples using metallic Cu-Cu and Cu-N/Cu-O paths as three-path fitting.

	Path	C.N.	R	dE	DW	R factor
Metallic Cu	Cu-Cu	12.0	2.54(2)	3.3(7)	0.007(1)	0.007
CuPc/CNP before CO_2R	Cu-N/O	3.8(1)	1.91(3)	-3.5(2)	0.007(3)	0.007
CuPc/CNP during CO_2R	Cu-N/O	0.6(3)	1.91(1)	-9.0(4)	0.003(1)	0.006
	Cu-Cu	6.7(2)	2.50(3)	-9.0(4)	0.009(4)	
EDTA/CuPc/CNP before CO_2R	Cu-N/O	3.6(1)	1.94(3)	-4.8(4)	0.003(1)	0.008
EDTA/CuPc/CNP during CO_2R	Cu-N/O	2.5(1)	1.89(1)	-2.4(3)	0.006(5)	0.006
	Cu-Cu	5.4(2)	2.56(3)	-2.4(3)	0.009(4)	

**Table S3 XPS integration areas for Cu 2*p* peaks and peak ratios of Cu(II):Cu(0)/(I)**						
	Cu(II) $2p_{3/2}$	Cu(0)/(I) $2p_{3/2}$	Cu(II) $2p_{1/2}$	Cu(0)/(I) $2p_{1/2}$	Peak ratio $2p_{3/2}$ Cu(0)/(I): Cu(II)	Peak ratio $2p_{1/2}$ Cu(0)/(I) Cu(II)
CuPc/CNP before CO_2R	20184.6	-	13841.7	-	-	-
CuPc/CNP after CO_2R	24201.5	41486.2	10368.8	22726.9	1.7	2.2
EDTA/CuPc/CNP before CO_2R	11611.8	-	5412.6	-	-	-
EDTA/CuPc/CNP after CO_2R	9590.1	11155.5	5116.0	5663.3-	1.2	1.1

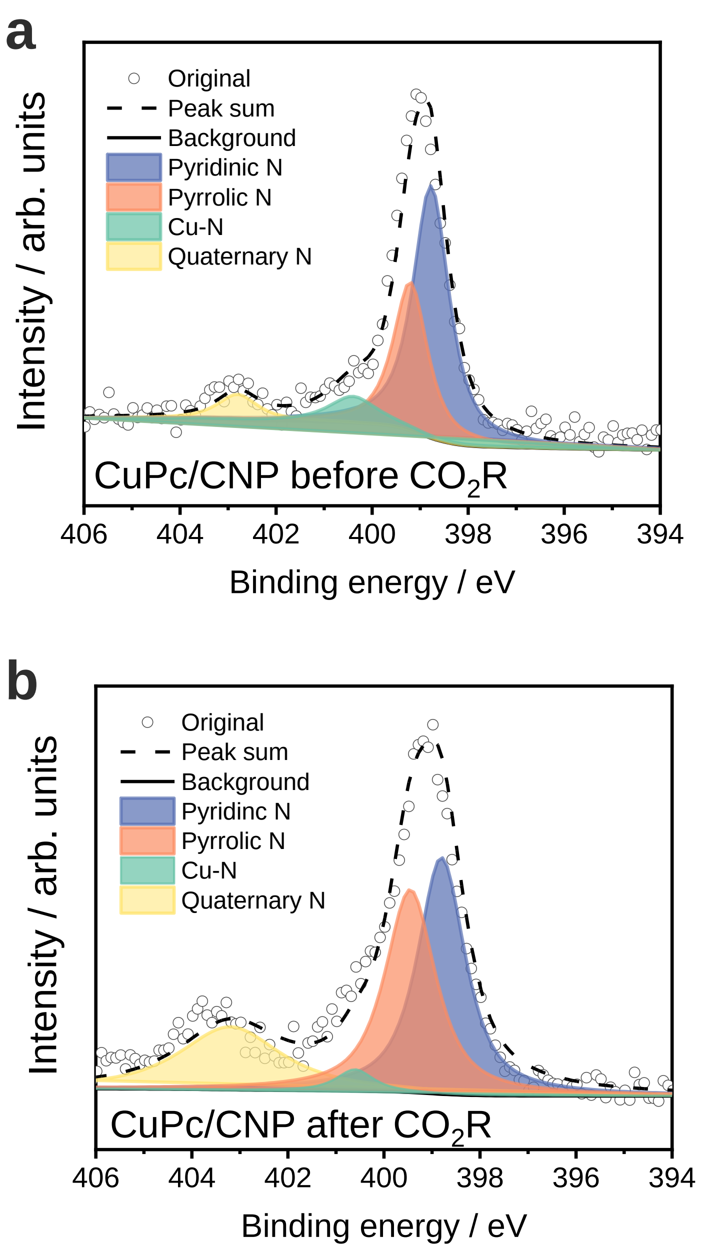

Figure S22 X-ray photoelectron spectra (XPS) high-resolution N 1*s* spectra of the CuPc/CNP samples. (a) Before CO_2R and (b) after CO_2R. For the post-electrolysis sample, we used N_2 to protect the cell when the current was cut off, and transferred the cell to a glove box. We disassembled the cell and stored the sample in a glove box to avoid contacting with O_2.

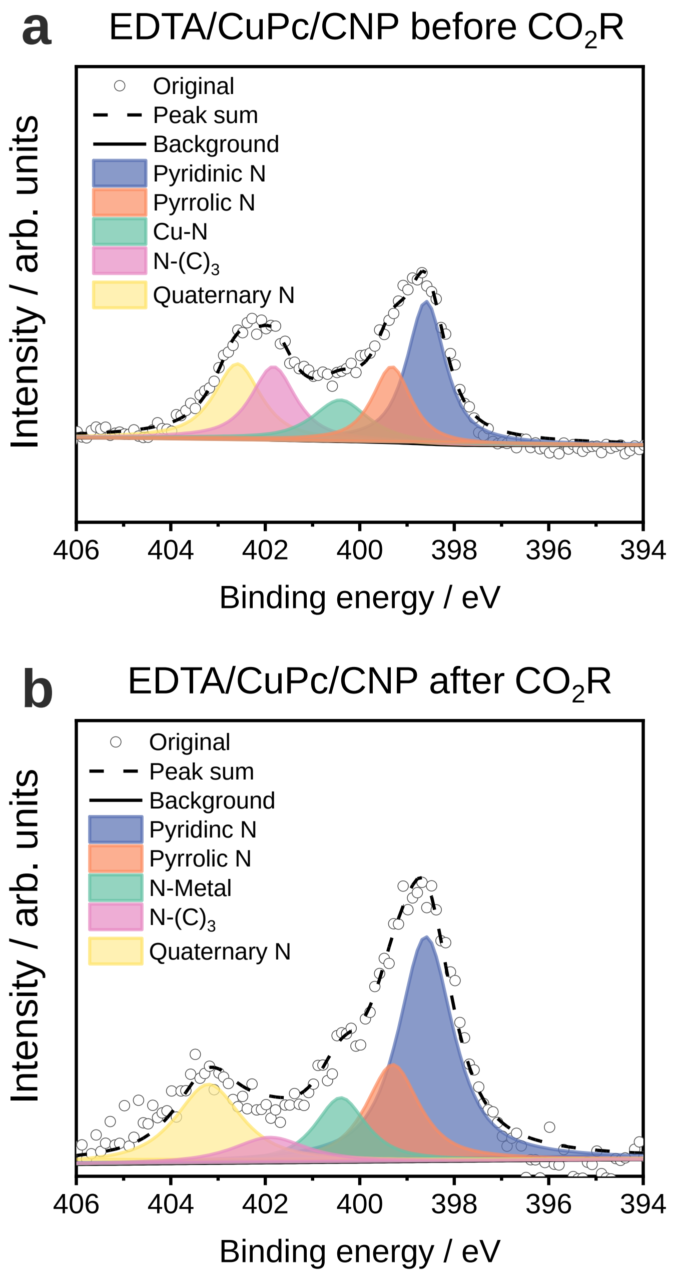

Figure S23 X-ray photoelectron spectra (XPS) high-resolution N 1*s* spectra of the EDTA decorated CuPc/CNP samples. (a) Before CO_2R and (b) after CO_2R. For the post-electrolysis sample, we used N_2 to protect the cell when the current was cut off, and transferred the cell to a glove box. We disassembled the cell and stored the sample in a glove box to avoid contacting with O_2.

Table S4 XPS integration areas for N 1*s* peaks. The Cu-N peak area normalized by the inert pyridinic peak area (which is assumed to not change during the reaction)

	Pyridinic N	Pyrrolic N	Cu-N	N-(C)$_3$	Quaternary N	Cu-N normalized value	Cu-N lost ratio
CuPc/CNP before CO_2R	18011.5	10011.5	2511.5	-	2011.5	0.14	57%
CuPc/CNP after CO_2R	12070.4	9047.6	770.6	-	6238.3	0.06	
EDTA/CuPc/CNP before CO_2R	12649.1	6780.6	5295.4	7558.6	8101.1	0.42	35%
EDTA/CuPc/CNP after CO_2R	4483.1	1875.8	1193.5	679.8	1889.2	0.27	

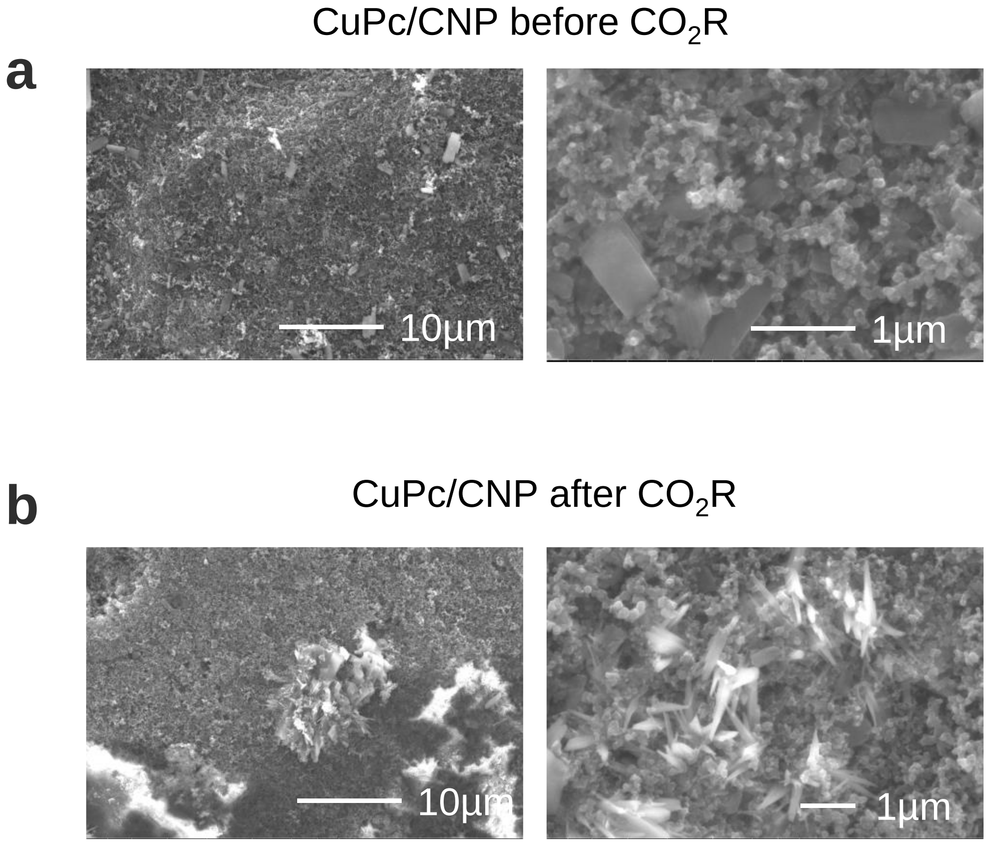

Figure S24 Scanning electron microscopy (SEM) and images of the CuPc/CNP sample. (a) before CO_2R, (b) after CO_2R. The CO_2R condition was under 100 mA cm^{-2} in an acidic MEA cell. All post-electrolysis samples were protected with N_2 before characterizations.

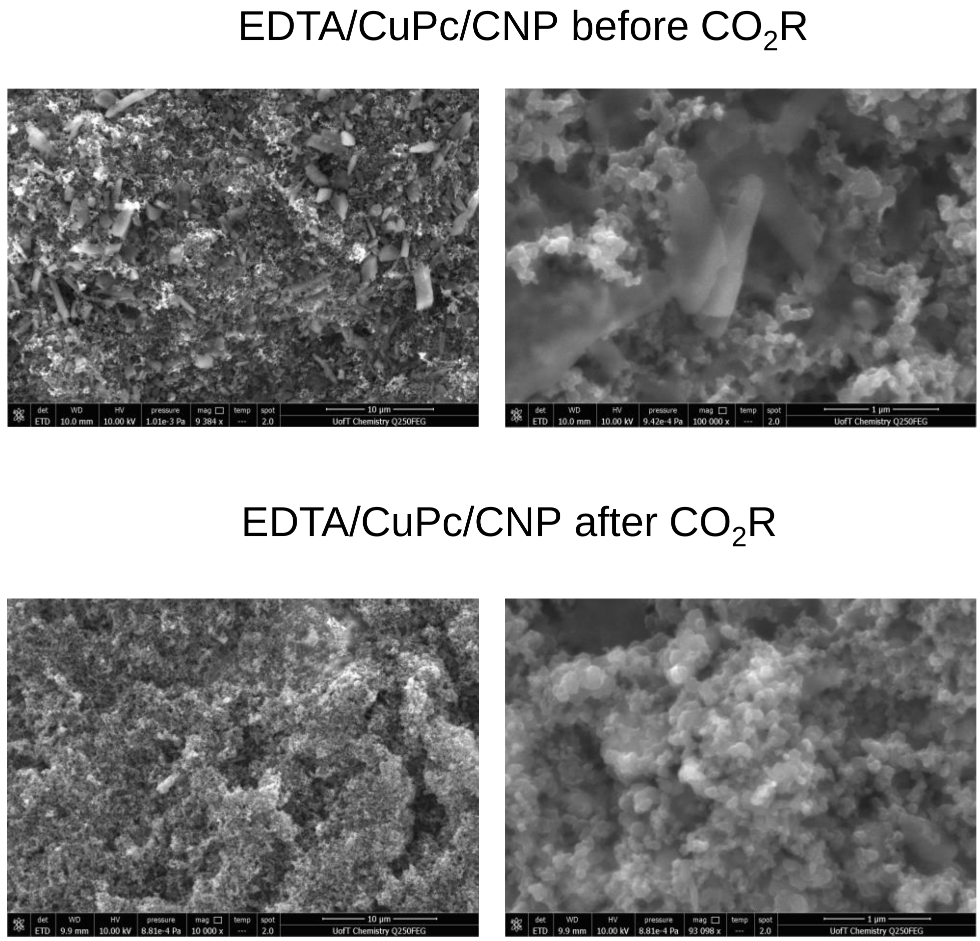

Figure S25 Scanning electron microscopy (SEM) and images of the EDTA decorated CuPc/CNP sample. (a) before CO_2R, (b) after CO_2R. The CO_2R condition was under 100 mA cm^{-2} in an acidic MEA cell. All post-electrolysis samples were protected with N_2 before characterizations.

References

1 Ozden, A. *et al.* Cascade CO_2 electroreduction enables efficient carbonate-free production of ethylene. *Joule* **5**, 706-719 (2021).

2 Xie, K. *et al.* Bipolar membrane electrolyzers enable high single-pass CO_2 electroreduction to multicarbon products. *Nat Commun* **13**, 3609 (2022).

3 Ma, M. *et al.* Insights into the carbon balance for CO_2 electroreduction on Cu using gas diffusion electrode reactor designs. *Energy Environ. Sci.* **13**, 977-985 (2020).

4 Sisler, J. *et al.* Ethylene electrosynthesis: A comparative techno-economic analysis of alkaline vs membrane electrode assembly vs CO_2–CO–C_2H_4 tandems. *ACS Energy Lett.* **6**, 997-1002 (2021).

5 Keith, D. W., Holmes, G., St. Angelo, D. & Heidel, K. A process for capturing CO_2 from the atmosphere. *Joule* **2**, 1573-1594 (2018).

6 Gao, H. *et al.* Comparative studies of heat duty and total equivalent work of a new heat pump distillation with split flow process, conventional split flow process, and conventional baseline process for CO_2 capture using monoethanolamine. *Int. J. Greenh. Gas Control.* **24**, 87-97 (2014).

7 Xu, Y. *et al.* Low coordination number copper catalysts for electrochemical CO_2 methanation in a membrane electrode assembly. *Nat. Commun.* **12**, 2932 (2021).

8 Zhang, L. *et al.* Enhanced cuprophilic interactions in crystalline catalysts facilitate the highly selective electroreduction of CO_2 to CH_4. *J. Am. Chem. Soc.* **143**, 3808-3816 (2021).
